# Interaction of Nitrate Assimilation and Photorespiration at Elevated CO_2_

**DOI:** 10.3389/fpls.2022.897924

**Published:** 2022-07-01

**Authors:** Konrad Krämer, Judith Brock, Arnd G. Heyer

**Affiliations:** Department of Plant Biotechnology, Institute of Biomaterials and Biomolecular Systems, University of Stuttgart, Stuttgart, Germany

**Keywords:** photorespiration, nitrate assimilation, elevated CO_2_, hydroxypyruvate reductase, *Arabidopsis*

## Abstract

It has been shown repeatedly that exposure to elevated atmospheric CO_2_ causes an increased C/N ratio of plant biomass that could result from either increased carbon or – in relation to C acquisition - reduced nitrogen assimilation. Possible reasons for diminished nitrogen assimilation are controversial, but an impact of reduced photorespiration at elevated CO_2_ has frequently been implied. Using a mutant defective in peroxisomal hydroxy-pyruvate reductase (*hpr1-1*) that is hampered in photorespiratory turnover, we show that indeed, photorespiration stimulates the glutamine-synthetase 2 (GS) / glutamine-oxoglutarate-aminotransferase (GOGAT) cycle, which channels ammonia into amino acid synthesis. However, mathematical flux simulations demonstrated that nitrate assimilation was not reduced at elevated CO_2_, pointing to a dilution of nitrogen containing compounds by assimilated carbon at elevated CO_2_. The massive growth reduction in the *hpr1-1* mutant does not appear to result from nitrogen starvation. Model simulations yield evidence for a loss of cellular energy that is consumed in supporting high flux through the GS/GOGAT cycle that results from inefficient removal of photorespiratory intermediates. This causes a futile cycling of glycolate and hydroxy-pyruvate. In addition to that, accumulation of serine and glycine as well as carboxylates in the mutant creates a metabolic imbalance that could contribute to growth reduction.

## Introduction

In the course of climate change a substantial increase of the atmospheric CO_2_ concentration is expected for the 21th century (IPCC, 2014; Szulejko et al., 2017). Because CO_2_ is the substrate for plant photosynthesis, alteration in the CO_2_ level have a direct impact on plant metabolism. The enzyme ribulose-1,5-bisphosphat-carboxylase/−oxygenase (Rubisco) catalyses CO_2_ fixation, but can also use O_2_ as substrate, resulting in the production of 2-phosphoglycolate, which is then further processed in the so-called photorespiration (PR) pathway (Shih et al., 2015). Because carboxylation is preferred over oxygenation (Sharkey, 1988), elevated CO_2_ concentrations (eCO_2_) will distinctly reduce the probability of oxygenation of Rubisco (Sharkey, 1988), and thus flux through the PR pathway will decline. Considering that a large proportion of the amino acids glycine (Gly) and serine (Ser) are produced during PR, this will affect plant primary metabolism.

In the course of PR two molecules of 2-phosphoglycolate (2-PG) are converted to one molecule of 3-phosphoglycerate, which is fed back into the Calvin-Benson-Cycle (Huma et al., 2018). More precisely, 2-PG is converted to glycolate which is subsequently oxidized to glyoxylate, producing H_2_O_2_ as a byproduct in peroxisomes. Next, glyoxylate is transaminated to Gly. The N source is either Ser or glutamate (Glu), resulting in the production of hydroxypyruvate and α-Ketoglutarate (α-KG), respectively (Nunes-Nesi et al., 2010). Hydroxypyruvate is reduced by the hydroxypyruvate-reductase (HPR) forming glycerate which can be phosphorylated to 3-phosphoglycerate (Timm et al., 2008). Gly is transported to mitochondria, where it is used by the glycine-decarboxylase (GDC), converting Gly, NAD^+^ and tetrahydrofolic acid to NADH, CO_2_, NH_4_^+^ and N^5^, N^10^ -methylene-tetrahydrofolic acid. The CO_2_ evolution in this step is eponymous for the PR (Rebeille et al., 1994). Together with N^5^, N^10^ -methylene-tetrahydrofolic acid a second molecule of Gly is converted to Ser by the enzyme serine-hydroxymethyl transferase (SHMT). Ser is transported to the peroxisomes, where it serves as N donor for Gly production from glyoxylate, yielding hydroxypyruvate (Nunes-Nesi et al., 2010). Besides energy consumption, the oxygenation of Rubisco and the subsequent PR create toxic intermediates such as 2-PG, glycolate and glyoxylate, which must be removed quickly (Anderson, 1971; Dellero et al., 2016). Even though the entire pathway, starting from oxygenation of ribulose-bisphosphate, appears wasteful, there is strong evidence that PR plays a role in the response to abiotic stress (Voss et al., 2013), for photoprotection (Guan et al., 2004) and nitrogen (N) assimilation (Cousins and Bloom, 2004; Bloom, 2015; Kraemer et al., 2021a).

Nitrogen assimilation mainly starts from nitrate that is reduced to nitrite and subsequently to ammonium by nitrate reductase (NR) and nitrite reductase, respectively (Xu et al., 2012). Ammonium is used to produce glutamine, the acid amide of Glu, catalyzed by glutamine synthetase (GS). The net synthesis of amino acids is then accomplished by the enzyme glutamine-oxoglutarate aminotransferase (GOGAT), which produces two molecules of Glu. Thus, the two enzymes GS and GOGAT create a cycle of Glu synthesis and amination that consumes ammonium, α-KG, ATP and reducing equivalents and yields Glu (Selinski and Scheibe, 2019).

Bloom (2015) proposed that increased PR flux causes higher malate levels in the cytosol. These could maintain turnover in the GS/GOGAT cycle and, concomitantly, produce NADH as substrate for NR. However, Andrews et al. (2019) showed that eCO_2_ affects N assimilation independently of the form of N administered, and that no inhibition of nitrate assimilation occurs. Thus, the mechanism by which eCO_2_ interferes with N assimilation remains unclear. Using a mathematical model that was parameterized by literature data, Zhao et al. (2021) demonstrated evidence that α-KG becomes limiting for N assimilation under eCO_2_, and we have obtained similar results investigating photosynthetic acclimation to eCO_2_ (Kraemer et al., 2021a). Because α-KG is central in the GS/GOGAT cycle, this again puts the PR pathway in focus. In this study we investigate the role of PR flux for N assimilation. We compare *Arabidopsis thaliana* plants grown at either ambient or eCO_2_. To create different PR fluxes, we used the *hpr1-1* mutant which lacks peroxisomal HPR (Timm et al., 2008) This results in elevated levels of Gly and Ser (Timm et al., 2021), stunted growth at ambient CO_2_ and a chlorotic phenotype (Li et al., 2019). The mutant shows elevated levels of α-KG and free amino-acids (Timm et al., 2021). We propose that growth reduction in the mutant is at least partly due to a disturbed energy household resulting from insufficient linear flux through PR.

## Materials and Methods

### Plant Growth and Photosynthesis Measurement

*A. thaliana* wildtype Col-0 and the mutant *hpr1-1* (SALK067724) were grown in hydroponic culture for 50 days in a growth chamber with 8 h/16 h light/dark regime (100 μmol m^−2^ s^−1^; 22°C/16°C). For the first 17 days plants were grown at ambient CO_2_ (450 ± 20 ppm). Afterwards half of the plants were transferred to eCO_2_ (1,000 ± 20 ppm). The hydroponic medium was as described in Brauner et al. (2014) with the difference that no ammonium was supplied and the nitrate concentration reduced to 2.175 mM. This assured sufficient, but not excess supply of N as identified in experiments with different levels of nitrate supply. For measurements of metabolites and enzyme activities, full rosettes of 50 day old plants were harvested every 2 h over a full diurnal cycle at fivefold replication under ambient as well as eCO2, resulting in a total of 120 samples. Photosynthesis measurements were conducted 1 week before harvesting as described by Küstner et al. (2019). Briefly, an infrared gas analysis system (Uras 3G; Hartmann and Braun AG, Frankfurt am Main, Germany) equipped with five custom made whole-rosette cuvettes and a sixth channel for measurement of CO_2_ in surrounding atmosphere was operated at a flow rate of 40 l/h, and each of the channels was measured sequentially for 6 min over a full diurnal cycle at a rate of 0.1 Hz. From the measured time points, splines were generated to yield a complete course of photosynthetic activity. All measurements were conducted at growth light and the respective CO_2_ concentration. Using the result of the photosynthesis measurements the photorespiratory activity was calculated according to the method developed by Sharkey (1988). For Γ* the values identified by Kraemer et al. (2021a) were used. As a first step, the ratio between oxigenation and carboxylation- Φ-was calculated according to equation (1).


(1) $$\text{Φ}=\left(2^{*}{\text{Γ}}^{*}\right)/\mathrm{Temperature}$$

Using this ratio the rate of oxygenation was calculated according to equation (2).


(2) $$v_{o}=\left(\mathrm{PS}–\mathrm{Respiration}\right)/\left(1/\text{Φ}–0.5\right)$$

The photorespiratory activity used in the model was calculated as the oxygenation rate divided by 2.

### Metabolite Measurements

Gly, Ser, α-KG, glucose, fructose and sucrose were measured by quantitative GC–MS/MS. Samples were extracted using 750 μl methanol with 25 nmol ribitol as internal standard. After 15 min at 70°C followed by shaking for 10 min at RT samples were centrifuged (5 min 17,000 g). The supernatant was transferred to a new vessel, and 400 μl of H_2_O were added. After incubation for 10 min at 95°C samples were agitated for 10 min at RT. Following centrifugation (5 min, 17,000 g) the supernatants were pooled. Subsequently, 300 μl H_2_O and 200 μl chloroform were added. After centrifugation (2 min, 17,000 g) the two phases were separated and the polar phase was dried in a speedvac and used for analysis. Dried samples were derivatized using 20 μl of methoxamine dissolved in pyridine (40 mg/ml) by incubation for 90 min at 30°C. Next, 80 μl N-methyl-N-(trimethylsilyl)trifluoracetamide (MSTFA) were added and the solution was incubated for 30 min at 50°C. Metabolites were measured by gas-chromatography coupled to mass-spectrometry (GC–MS/MS). For injection, 1 μl of the derivatized sample was used. The GC–MS/MS device was a GCMS-TQ8040 (Shimadzu, Kyoto, Japan) using helium as carrier gas at a flow of 1.12 ml/min. The stationary phase was a 30 m Optima 5MS-0.25 μm fused silica capillary column. Injection temperature was 230°C. The transfer line and ion source were set to 250°C and 200°C, respectively. The initial temperature of the column oven was 80°C and this was increased by 15°C/min until the final temperature of 330°C was reached and held for 6 min. After a solvent delay of 4.6 min, spectra of the MS device were recorded in the multiple reaction mode (MRM) with specific target-ions for each metabolite. External standards were used for quantification.

Starch, hexose-phosphates and the total amino-acid pool were determined photometrically and carbonic acids (fumaric acid, malic acid and citric acid) by HPLC as described by Küstner et al. (2019). Ammonium was quantified according to Vega-Mas et al. (2015). Glu and Gln were measured according to Graham and Aprison (1966) and Pérez-de la Mora et al. (1989).

### Enzymatic Activities

Activity of nitrate reductase was measured as described by Scholl et al. (1974). Activity of hydroxy-pyruvate-reductase was determined according to Bauwe (2017). GS activity was measured according to Berteli et al. (1995) and Silveira et al. (2003). Briefly, protein was extracted into a 100 mM Tris–HCl (pH 7.6) buffer containing 2.5 mM dithiothreitol and 10 mM MgCl_2_. The assay buffer contained 125 mM Tris–HCl (pH 7.6), 5 mM ATP, 80 mM MgSO4, 125 mM hydroxylamine-NaOH (pH 7) and 100 mM glutamate (pH 7.2). To an assay buffer volume of 80 μl, 120 μl of protein extract were added and incubated for different time-points (0, 20, 25 and 30 min). To determine the background, an assay buffer was used without hydroxylamine-NaOH (pH 7). The reaction was stopped using 60 μl of a solution consisting of 1.5 ml 10% w/v FeCl_3_*6 H2O in 0.2 N HCl, 1.5 ml 24% w/v trichloroacetic acid and 1.5 ml 20% v/v HCl. Afterwards, the absorption was determined at 540 nm. For calibration curves L-glutamic acid γ-monohydroxamate was used.

All enzyme activities were determined at the beginning, middle and end of the light phase as well as in the middle of the night. Values for the remaining time-points were calculated by spline interpolation. For the calculation of reaction velocity, substrate concentration was calculated from the measured metabolite contents, assuming that 1 g of plant biomass had a volume of 0.77 ml.

### Data Analysis and Statistics

Data evaluation, normalization, visualization and statistics were performed in Microsoft Excel (RRID:SCR_016137) and the R software (R Project for Statistical Computing, RRID:SCR_001905). The experimental design was a randomized complete block design with CO_2_ level as block and genotype as treatment. If not otherwise stated, two-way ANOVA for genotype and CO_2_ treatment effects were used for statistical analysis. Parameter optimization was performed in a way that *in silico* time courses best matched the measured time courses. For optimization and simulations the R-package paropt was used (Kraemer et al., 2021b).

## Results

### Diurnal Dynamics of Metabolites

Net photosynthetic rates (*PS*) increased by about 50% in the wildtype, when CO_2_ concentration was raised to 1,000 ppm. The wildtype had significantly higher *PS* than the mutant (*p* < 2e-16) under both conditions (Col-0 at ambient CO_2_ = 85.4 ± 12.8 μmol/g * h, Col-0 at eCO_2_ = 128.1 ± 44.9 μmol/g * h, *hpr1-1* at ambient CO_2_ = 76.8 ± 19.9 μmol/g * h, *hpr1-1* at eCO_2_ = 98.6 ± 35.8 μmol/g * h). An increase in net photosynthesis by about 50% at eCO_2_ in the wildtype is in accordance with previous observations (Jauregui et al., 2016). However, a stronger reduction of photosynthesis in *hpr1-1* was reported for higher light intensities by Timm et al. (2008).

Despite the lower *PS* rate and no obvious deviations in hexose-phosphate levels (Supplementary Image 1), the combined pool of malate and fumarate (MF) was larger in the *hpr1-1* mutant than in wildtype (*p* = 4.1e-10). Citrate (Cit) was elevated in *hpr1-1* only at ambient CO_2_ (Supplementary Image 1; *p* = 2.47e-7) and showed a different response to rising CO_2_ levels: while it increased in the wildtype (*p* = 7.1e-4, *t*-test for wildtype), it declined in the mutant (*p* = 5.08e-6, *t*-test for *hpr1-1*).

**Figure 1:**
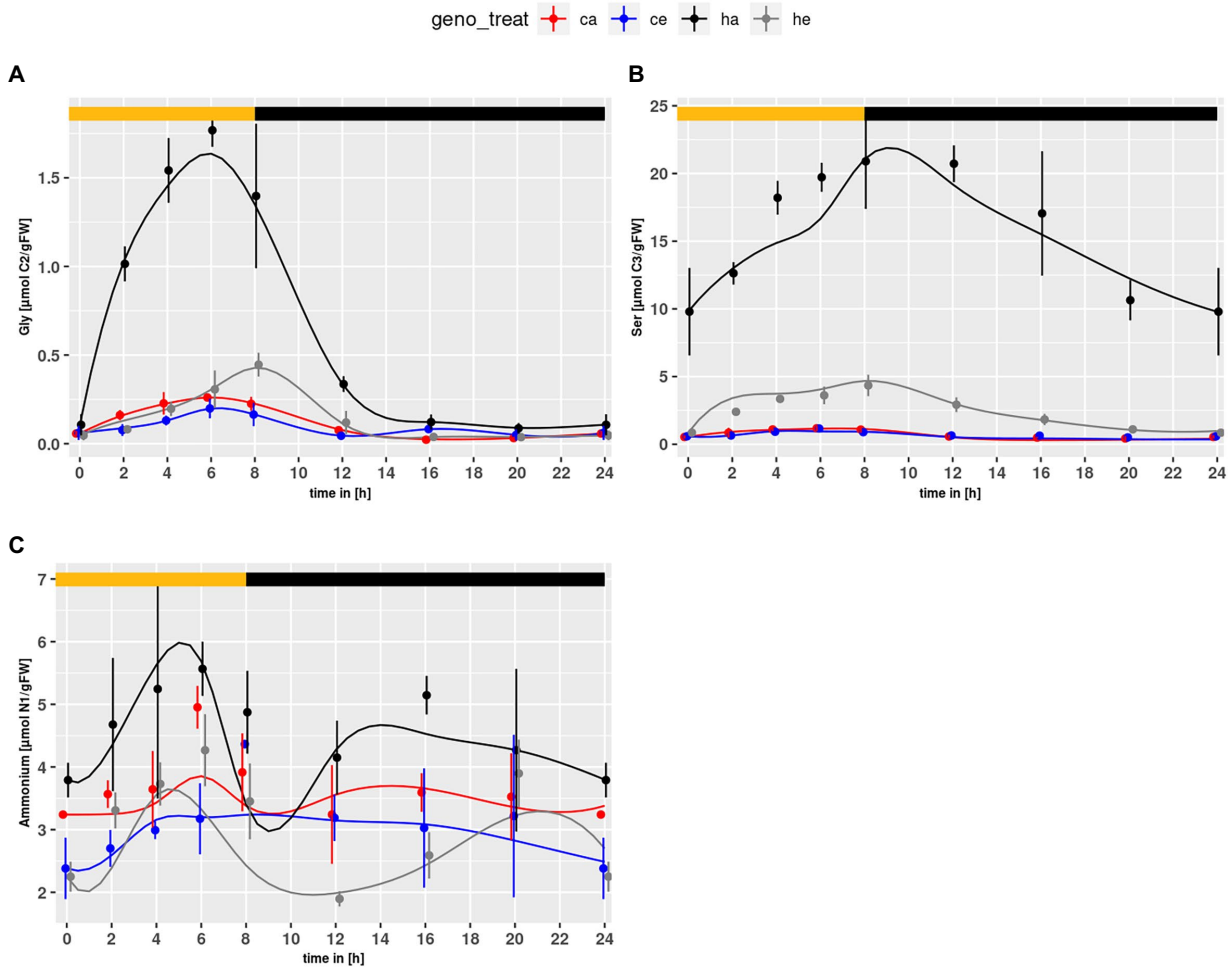
Diurnal course of photorespiratory intermediates; **(A)** glycine, **(B)** serine, **(C)** ammonium. Col-0 ambient: red, Col-0 eCO_2_: blue; *hpr1-1* mutant ambient: black, *hpr1-1* mutant eCO_2_: grey. Shown are means with standard error (*n* = 5). Lines represent the mean of 20 simulations. Light phase is indicated by yellow bar and dark phase indicated by black bar.

The PR intermediates Gly and Ser accumulated in the mutant under both conditions (Figures 1A,B). As expected, the contents were higher at ambient CO_2_. For NH_4_^+^, which is also produced during PR, significant genotype and treatment effects were observed with higher levels in *hpr1-1* and a general decrease under eCO_2_ (*p* = 5.67e-5 and *p* = 4.38e-6, respectively).

**Figure 2:**
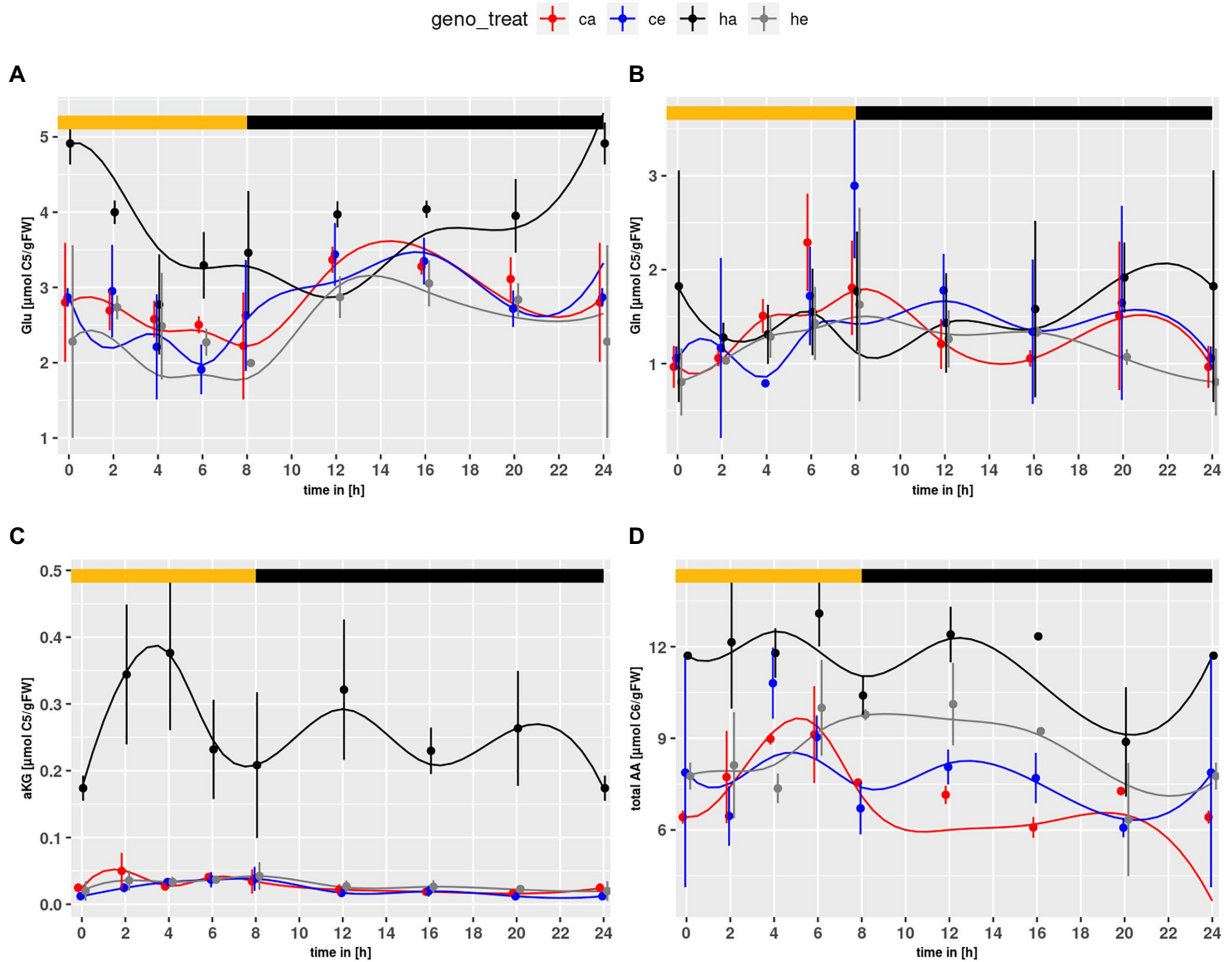
Diurnal course of **(A)** glutamate, **(B)** glutamine, **(C)** α-ketoglutarate, **(D)** total amino acids. Col-0 ambient: red, Col-0 eCO_2_: blue; *hpr1-1* mutant ambient: black, *hpr1-1* mutant eCO_2_: grey. Shown are means with standard error (*n* = 5). Lines represent the mean of 20 simulations. Light phase is indicated by yellow bar and dark phase indicated by black bar.

Similar to the NH_4_^+^ content, Glu levels (Figure 2A) were elevated in the mutant as compared to wildtype (*p* = 7.71e-4) and significantly increased in mutant plants at ambient CO_2_ as compared to eCO_2_ (*p* = 2.03e-10). This effect could not be observed for Col-0. Neither genotype nor treatment effects were observed for Gln (Figure 2B). However, when considering the mutant alone, an elevated level at ambient CO_2_ became obvious (*p* = 0.0163). The α-KG content (Figure 2C) was strongly increased for *hpr1-1* at ambient CO_2,_ Considering that Glu is converted to α-KG when used as substrate for transamination of glyoxylate in the peroxisome, elevated α-KG may point to an increased Glu turnover for transamination of glyoxylate.

Furthermore, the amino acid (AA) pool (Figure 2D) was elevated in the mutant compared to wildtype (*p* < 2.0e-16). Considering only mutant data, an increase at eCO_2_ was significant (*p* < 2.0e-16). Thus, no indications for N-starvation in the mutant were found.

### Diurnal Dynamics of Parameters for Enzyme Activity

Maximum activities were determined for three enzymes central to *de novo* N assimilation, amino acid metabolism and PR. For NR, the Col-0 wildtype showed the highest activity at eCO_2_ (Supplementary Image 2A). This constituted a significant genotype effect (*p* = 0.0346) that was unexpected considering reports of decreased N assimilation at eCO_2_ (Bloom et al., 2014). In addition, a time effect with highest activity at midday was observed independent of the condition (*p* = 0.0017). GS activity (Supplementary Image 2B) was clearly affected by the CO_2_ level with higher activity at ambient CO_2_ than at eCO_2_ (*p* = 4.14e-13). Notably, *hpr1-1* had even higher *GS* activity than Col-0 when grown at ambient CO_2_ (*p* = 0.0021), indicating a higher potential for turnover in the GS/GOGAT cycle. Not surprisingly, a genotype effect was found for HPR activity (Supplementary Image 2C; *p* < 2e-16). In addition, a treatment effect was detected, which was dependent on the *hpr1-1* data (*p* = 0.0461).

### Model Construction

**Figure 3:**
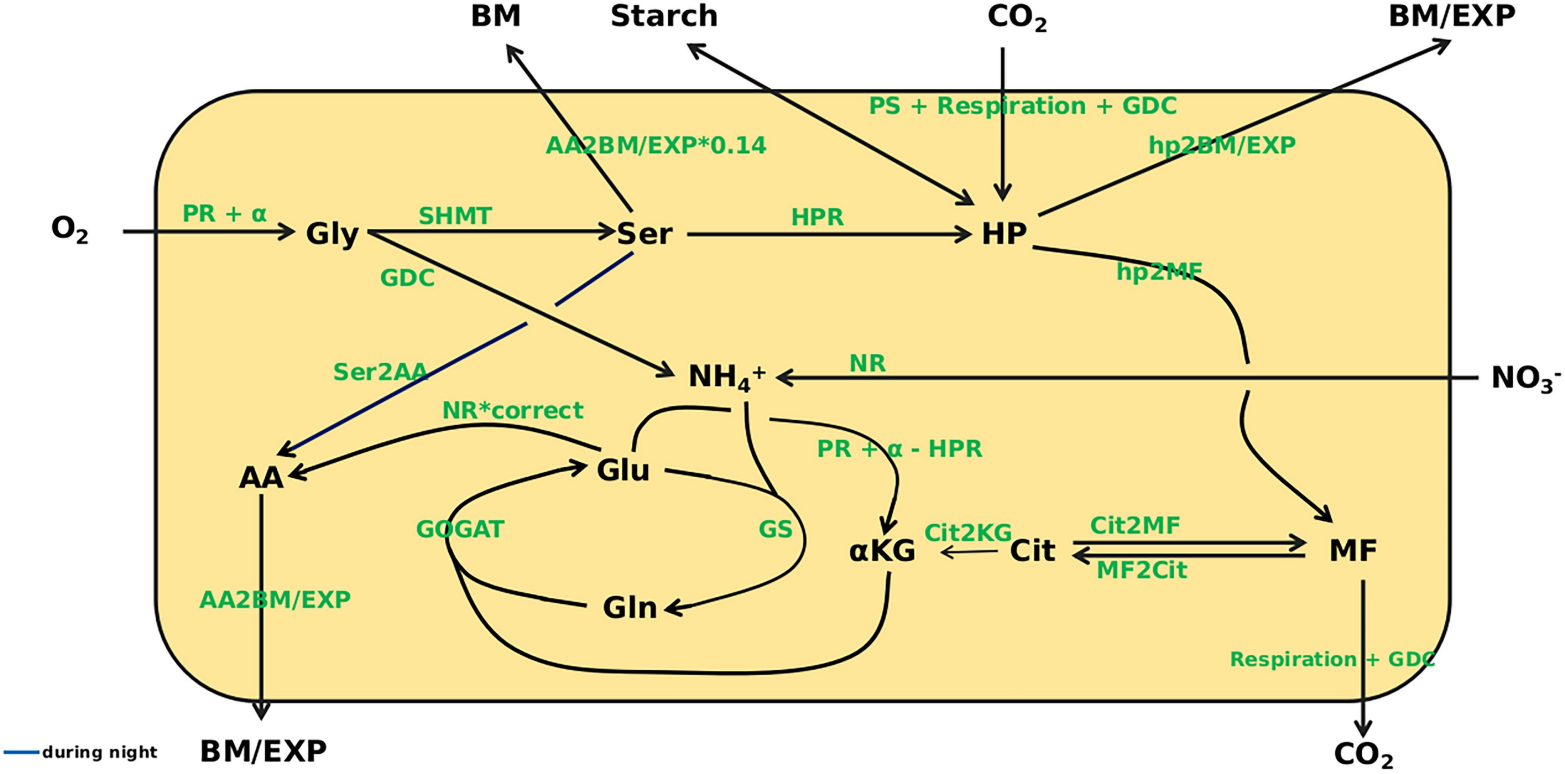
Overview of the model. Model states are given in black. O_2_, CO_2_, biomass (BM), Starch, export (EXP), and NO_3_^−^ are outside the system boundary. Gly, glycine; Ser, serine; HP, hexose-phosphates; MF, malate + fumarate; Cit, citrate; α-KG, α-ketoglutarate; Glu, glutamate; Gln, glutamine; AA, amino acids; NH_4_^+^, ammonium. For the following fluxes mass balance kinetics were used: hp2BM/EXP, hp2MF, Cit2MF, MF2Cit, Cit2KG, Ser2AA, AA2BM/EXP. The remaining reactions are represented as Michaelis–Menten kinetics.

With the aim of mathematically simulating C/N interactions at eCO_2_, a dynamic model based on ordinary differential equations (ODE) was constructed that covered carbon as well as nitrogen acquisition. The model structure is depicted in Figure 3. Based on measurements of photosynthesis and respiration as well as metabolite content at 8 time points over a diurnal cycle, kinetic parameters were identified that allowed simulation of metabolite dynamics with only the metabolite levels at the beginning of the light period (*t* = 0) given. The identified parameters (see Supplementary Table 1) describe turnover rates for C- and N-compounds. Besides concentrations of O_2_ and CO_2_, the plant biomass (BM), starch level, amount of assimilates exported from the leaves (EXP) and nitrate level are regarded as beyond the system boundaries, which means that, e.g., the rate of starch synthesis is included in simulations, but not the actual amount that has accumulated at a certain time point. In contrast, levels of Gly, Ser, HP, MF, Cit, α-KG, Glu, Gln, AA and NH_4_^+^ are included in the simulations. For the carbon fluxes to biomass and sink organs (*hp2BM/EXP*; note: all fluxes in italics), to or among carboxylic acids (*hp2MF*, *Cit2MF*, *MF2Cit*, *Cit2KG*), as well as the C/N fluxes from serine to amino acids (*Ser2AA*) and amino acids to biomass and export (*AA2BM/EXP*), mass balance kinetics were applied. The remaining reactions are represented as Michaelis–Menten kinetics. Kinetic equations for enzyme reactions are given in file Supplementary Data Sheet 1. The full ODE system is given in file Supplementary Data Sheet 2.

Initial steps of the PR pathway are represented by a reaction yielding Gly, which means that all reactions from oxygenation of ribulose-bisphosphate to glyoxylate formation were not resolved, because absolute quantification of the respective intermediates is not reliable. Input into the pathway was calculated on the basis of *PS* according to Sharkey (1988). Gly is converted to Ser, which was considered precursor for the *HPR* reaction that feeds back into the pool of sugar-phosphates. Alternatively, Ser can flow into the pool of the other AA, representing the reaction of Ser-pyruvate aminotransferase.

All short-lived intermediates of the Calvin-Benson-Cycle, triose-phosphates and hexose-phosphates were represented by the HP pool, which is quantitatively dominated by glucose-6-phosphate and fructose-6-phosphate. The latter were quantified enzymatically (see Material and Methods). Photosynthesis, measured as CO_2_ uptake per time, constituted the carbon input into this pool. Measured values were corrected for mitochondrial respiration and PR input. Mitochondrial respiration was measured during the dark phase and set constant over the entire diurnal cycle as earlier suggested by Küstner et al. (2019) and Kraemer et al. (2021a). The HP pool was set as the precursor for cell wall synthesis and the supply of carbon to sinks. It should be mentioned that the transport metabolite, sucrose, attains much higher levels than measured for HP, which is substrate for cell wall biosynthesis. While this would affect the ratio of BM formation and transport rates, when kinetic parameters are constrained, this was not the case in the current model, which left wide ranges for the BM/EXP parameter, because assimilate export was not in focus. In the model, assimilate export and biomass formation were represented by the combined flux *hp2BM/EXP*. In addition, HP is in exchange with starch that serves as carbon source during the night. Starch production during the day and degradation at night was assumed to be linear as outlined by Stitt and Zeeman (2012). As demonstrated by Gauthier et al. (2010) and Tcherkez et al. (2009), Cit is not, or only to a very low extent, produced during the day, but accumulates during the night and is used for α-KG production during the next day (Cheung et al., 2014). Therefore, the substrate for accumulation of MF during the light phase was considered to be HP, thus referring to anaplerotic reactions. However, the possibility of a flux from Cit to MF was not excluded. MF is used for mitochondrial respiration.

During the reaction from Gly to Ser NH_4_^+^ is produced and, together with Glu, can be used by GS to produce Gln (Supplementary Image 2). Gln can transaminate α-KG, catalyzed by GOGAT, to produce two molecules of Glu. Two reactions can yield α-KG that is required for AA production. The first reaction is from Cit (Cheung et al., 2014) representing the TCA cycle and cytosolic isocitrate dehydrogenase activity (Hodges, 2002). The second is from Glu and includes the reaction of Glu-glyoxylate-aminotransferase, which is part of PR, as well as mitochondrial Glu dehydrogenase and transamination reactions involving Glu. For Gly production, a source of N is required. This can derive from Ser, which, in the model, is included in the combined *HPR* flux. Alternatively, Glu can serve as N donor for Gly production. The *PR* flux into the system, the *HPR* flux, and the reaction from Glu to α-KG are all N_1_-fluxes. Thus, the three fluxes can be balanced, and Glu to α-KG can be expressed as *PR* minus *HPR*.

Another source of NH_4_^+^ is the reduction of nitrate by NR (Supplementary Image 2). Modeling this *de novo* N fixation is complicated by the fact that a large proportion of nitrate is stored in the vacuole and not accessible by NR. To account for this compartmentation, the content was adjusted based on the cellular proportion of cytosol, which is about 5% of total cell volume in mature leaves (Koffler et al., 2013). *NR* is the only flux providing *de novo* assimilated N. Thus, the step towards AA production was linked to *NR*. According to Gauthier et al. (2010) about 50% of Ser consist of newly assimilated N. Accordingly, we modeled the flux from Glu to AA as *NR* multiplied by the correction factor, *correct*, which was set in the interval [0.5, 1]. Because the PR intermediates Gly and Ser are the only other sinks for *de novo* assimilated N in our model, *correct* likewise determines the deposition of new N in these compounds.

Because *PR* flux, as defined by Sharkey (1988), is directly coupled to photosynthetic activity, the pathway would not operate in the dark, which would, in our model, exclude turnover of Gly and Ser after light-off. However, especially in the case of *hpr1-1* at ambient CO_2_, Ser turnover was substantially extended into the night. This was accounted for by adding the summand α to the formula for *PR*, which allows operation of pathway reactions described by Walton and Butt (1981) independent of photosynthetic activity.

The step from Gly to Ser is catalyzed by the two enzymes *GDC* and *SHMT*. For both reactions Michaelis–Menten kinetics were employed. However, turnover of *SHMT* cannot exceed that of *GDC*, and thus *SHMT* was limited to the *GDC* value. In contrast, it is known from the literature that the *GDC* flux may well be substantially higher than the flux of *SHMT* (Rebeille et al., 1994).

Because the model structure presented in Figure 3 does not allow the amino acids Gly and Ser to contribute to biomass *via* the *AA2BM/EXP* route, a separate pathway was established for these compounds. Based on proteome information for *Arabidopsis* (Berardini et al., 2015) a proportion of about 10% of the amino acids in protein should be Ser. However, Rubisco, which makes up about one third of total protein (Atkinson et al., 2017), has 14% Ser, and thus, the flux from Ser to BM was set to 14% of the flux from AA to BM/EXP.

### Preliminary Parameter Identification

**Figure 4:**
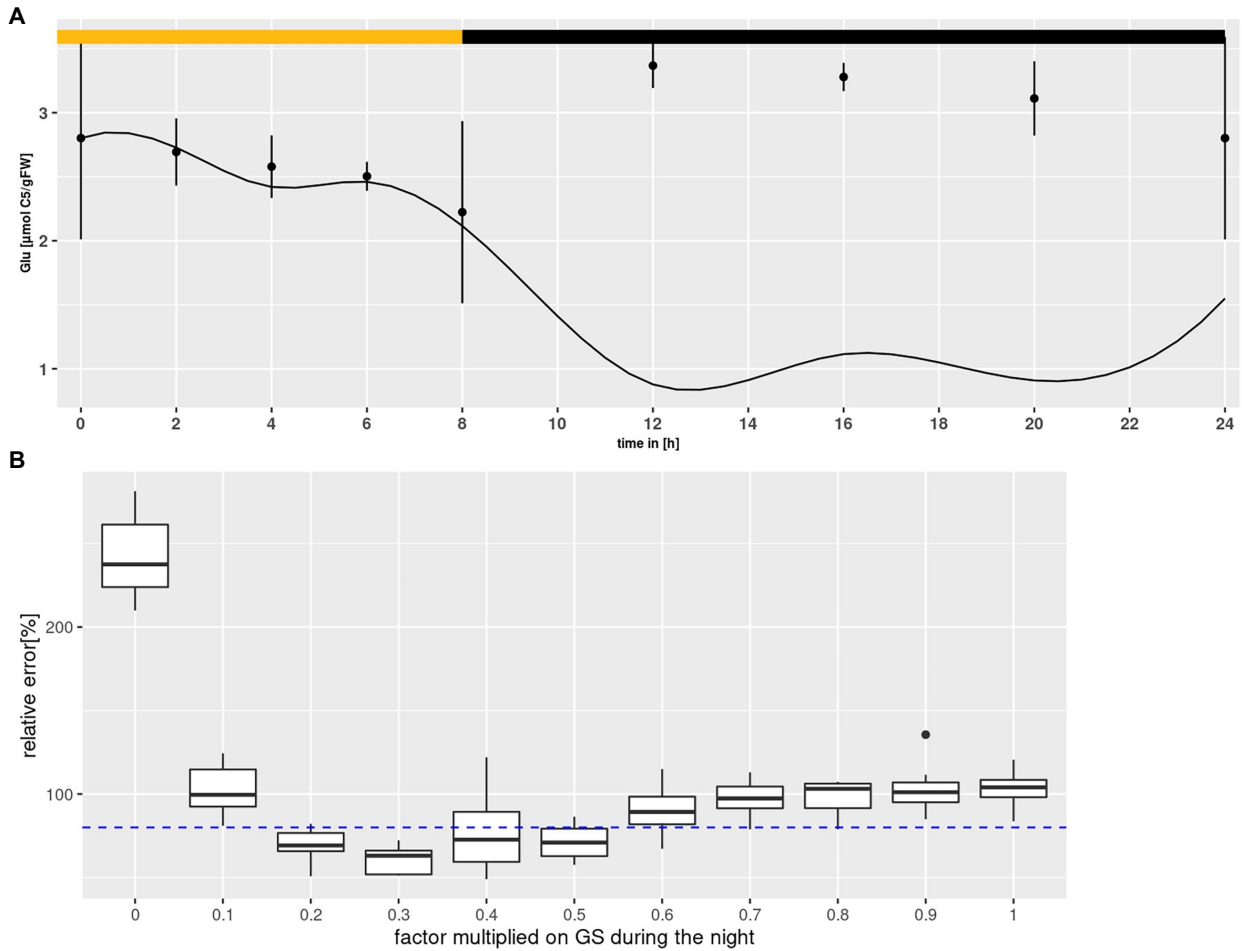
Glutamine synthetase (GS) inactivation. **(A)** Diurnal course of glutamate for Col-0 at ambient CO_2_ concentrations. Shown are mean with standard error (*n* = 5). Lines represent the mean of 10 simulations. Light phase indicated by yellow bar and dark phase indicated by black bar. **(B)** Error plotted against the factor multiplied with GS activity during the night. Each boxplot represents 10 simulations for Col-0 at ambient CO_2_ concentrations. The dashed line indicates the threshold at which results for simulations of all states were accepted (see text for criteria).

According to the above described model structure several rounds of parameter optimization were conducted that yielded results for most states that were covered by the measured standard deviation with an averaged error between 0.05 and 0.1% per state and time point. However, Glu levels were consistently underestimated, particularly during the night as shown in Figure 4A. Choi et al. (1999) reported that the GS enzyme of *Canavalina lineata* is activated by a reduction at two cysteine residues, which are conserved among all known plastidial GS sequences. The authors demonstrated that reductants like dithiothreitol increase the activity of the plastidial isoform. Considering that dithiothreitol was added during protein extraction (see “Materials and Methods”), it is very likely that GS activity was overestimated, especially during the night. It is known that, following light-off, the redox milieu of the chloroplasts changes rapidly (Dietz and Hell, 2015). However, the extent of change in GS activity is unknown. Therefore, a numerical experiment was set up to mathematically assess GS activity during the night (see Figure 4B). Nocturnal GS activity was intentionally reduced in steps of 10%, and 10 simulations of model states were conducted for each step. Simulations were accepted, when results for all states lay within the measured standard deviation. As can be seen in Figure 4B, the cumulative error of simulations was minimized for nocturnal GS activity between 20 and 50% of the *in vitro* activity. This agrees with the data of Choi et al. (1999), who showed that dithiothreitol increased the activity by about two-fold. Thus, we added a factor in the model by which *GS* can be down-regulated by the optimizer during the night.

### Photorespiration in the *hpr1-1* Mutant

As can be seen from the model structure in Figure 3, fluxes such as *HPR* summarize multiple enzymatic steps, for which kinetic parameters may deviate from those determined for individual enzymes that are rate-limiting under most conditions. Thus, for identification of the k_m_ of the HPR reaction a broad interval was set in order to integrate activity of several enzymes and transporters, for which no parameter boundaries are known. This includes transport of Ser out of the mitochondria and into the peroxisomes, its de-amination by Ser-glyoxylate transaminase, the actual reduction by HPR and finally the phosphorylation of glycerate before its re-integration into the Calvin-Benson cycle. Most importantly, it is unclear whether in Col-0 HPR constitutes the rate-limiting step in the PR pathway.

**Figure 5:**
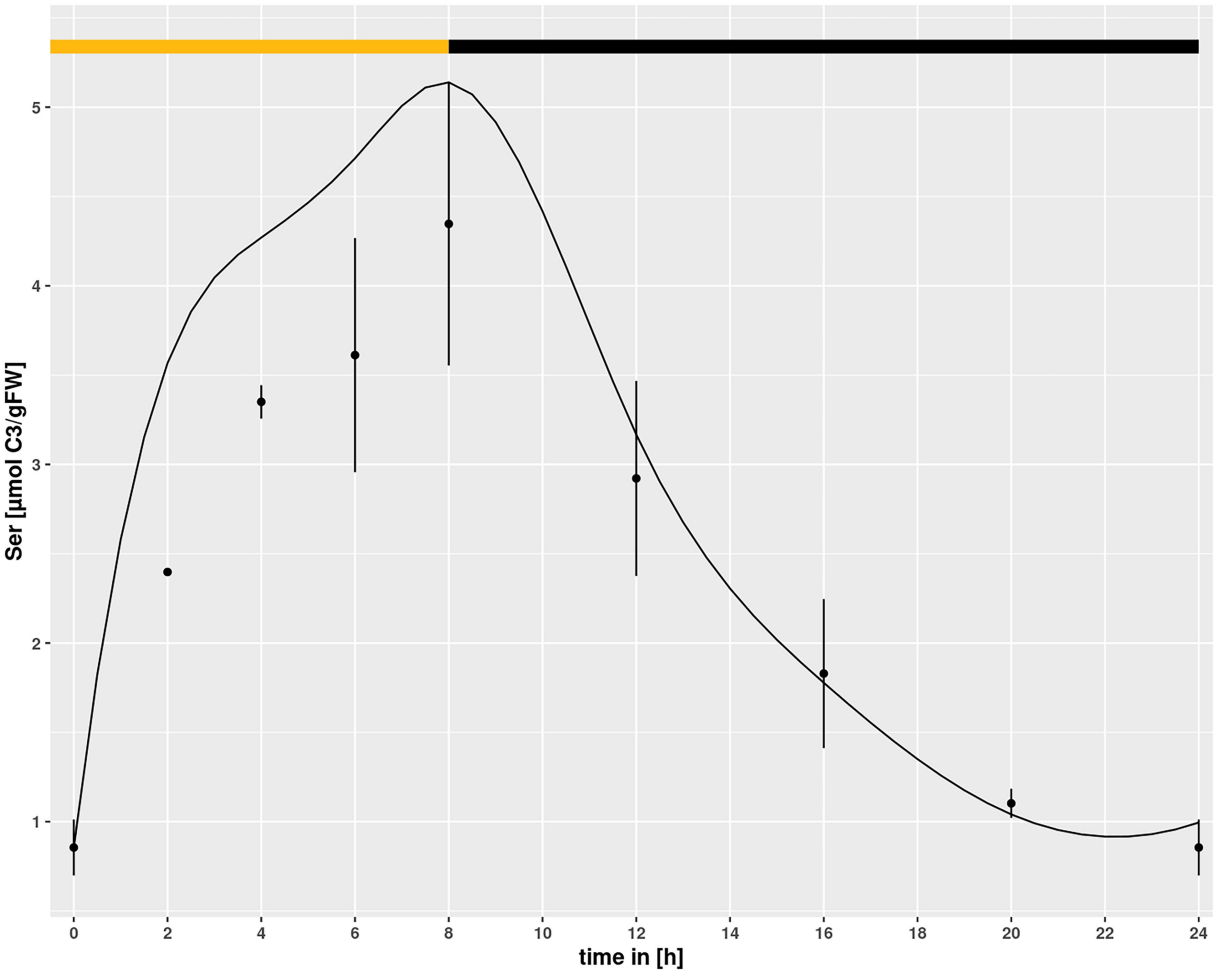
Diurnal course of serine for *hpr1-1* at eCO_2_ concentrations. Dots represent means with standard error (*n* = 5). Lines represent the mean of 20 simulations using the minimum of the km value of the simulations of Col-0 ambient CO_2_ as lower boundary. The light phase is indicated by a yellow bar and dark phase indicated by black bar.

Setting a broad interval for the *HPR* parameters created a problem for simulations in *hpr1-1*, because it allowed for a purely mathematical compensation of the low v_max_ in the mutant by choosing an adequately low k_m._ To prevent this, the identified minimum of the k_m_ value for HPR in Col-0 was set as lower boundary for simulations of the *hpr1-1* mutant with the underlying assumption that the k_m_, being a feature of a protein, cannot easily change. However, using this approach the simulated Ser pool was slightly higher then measured for the mutant at eCO_2_ (Figure 5) albeit not at ambient. Opening of the lower boundary to allow a further 30% reduction of the k_m_ could correct this, as demonstrated by the simulations in Figure 1B, and was thus used as setting for all simulations.

### Observed Flux Dynamics

**Figure 6:**
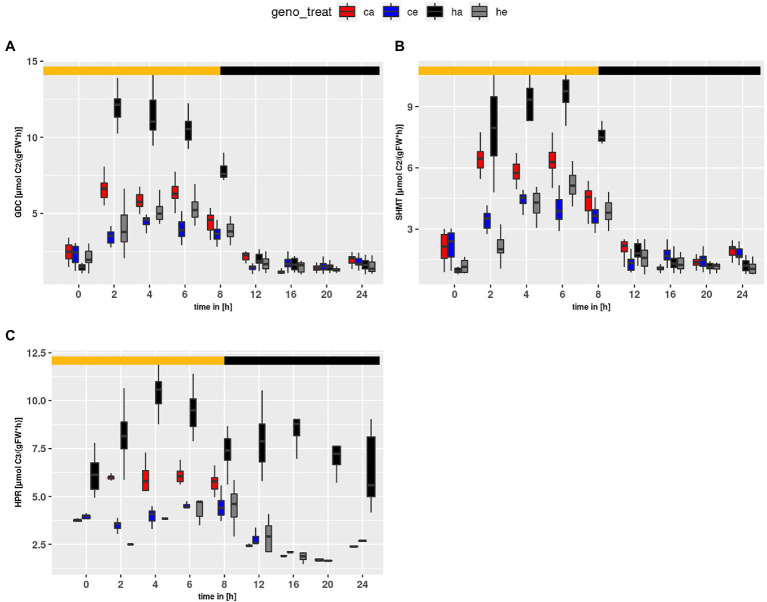
Diurnal course of fluxes for **(A)** *GDC*, **(B)** *SHMT* and **(C)** *HPR*. Col-0 ambient: red, Col-0 eCO_2_: blue; *hpr1-1* mutant ambient: black, *hpr1-1* mutant eCO_2_: grey. Shown are means of results for 20 simulations. Light phase is indicated by yellow bar and dark phase indicated by black bar. Boxplots are dodged by 0.25 h in order to prevent overlap.

Using the parameter sets obtained from the simulations, flux rates at time points of harvest were calculated. Figure 6 shows calculated flux rates for PR-relevant reactions. As expected, *HPR* flux was higher at ambient than eCO_2_ (*p* < 2e-16) due to the larger PR input. Similar, *GDC* and *SHMT* (Figures 6A,B) were increased at ambient CO_2_ as compared to eCO_2_ (*p* < 2e-16). Surprisingly, *HPR* (Figure 6C) showed higher flux rates in the mutant as compared to wildtype (*p* < 2e-16). This resulted from extremely high levels of Ser, which is the substrate in our compiled HPR reaction, and indicated a metabolic state clearly different from wildtype. For instance, the Ser levels already at the beginning of the day are substantially elevated in the mutant at ambient CO_2_. Moreover, in comparison to wildtype the *hpr1-1* mutant showed increased levels for *GDC* (*p* = 1.16e-10). In contrast, the *SHMT* reaction tended to be increased in the wildtype, though this was not significant.

**Figure 7:**
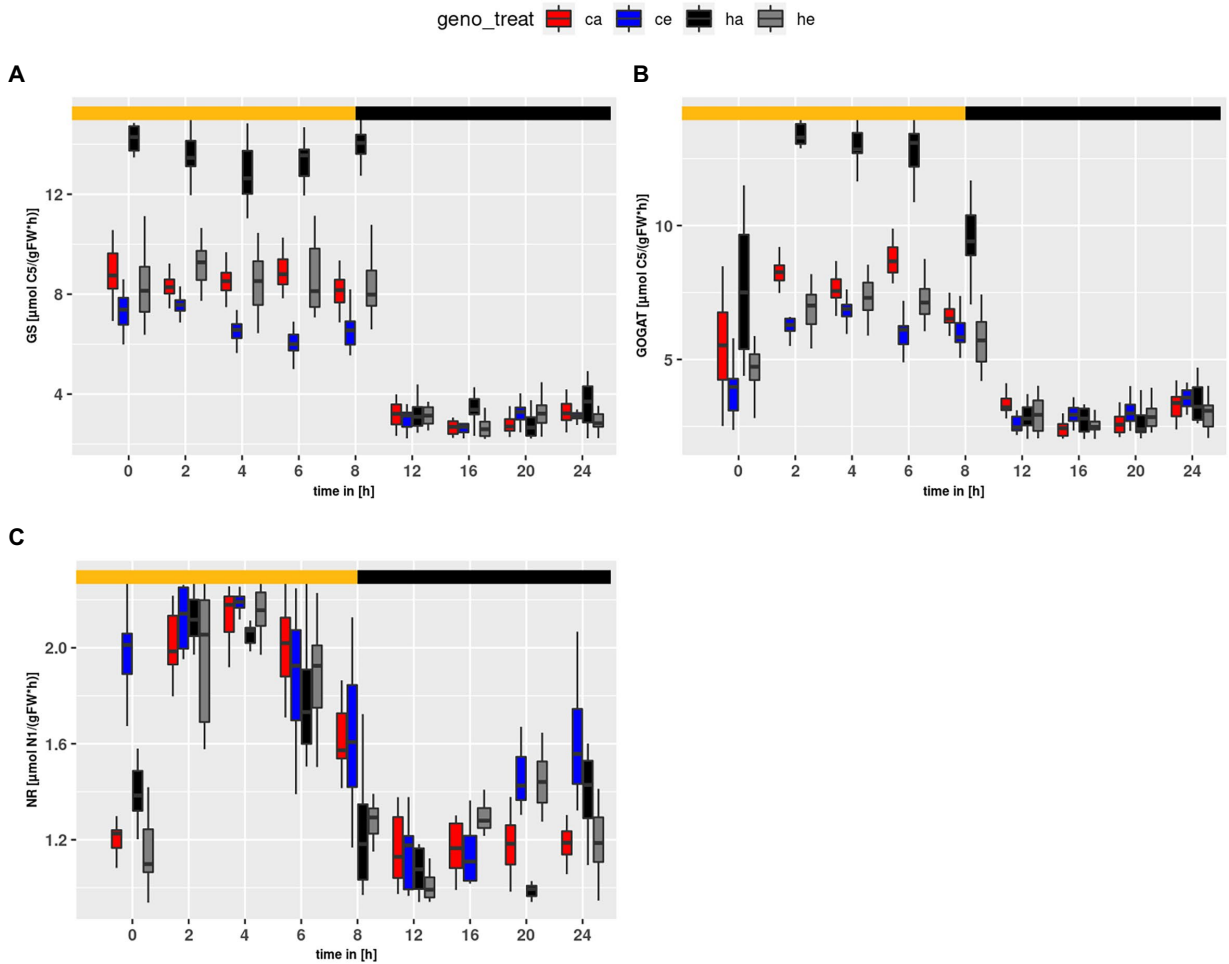
Diurnal course of fluxes for **(A)** GS, **(B)** GOGAT and **(C)** NR. Col-0 ambient: red, Col-0 eCO_2_: blue; *hpr1-1* mutant ambient: black, *hpr1-1* mutant eCO_2_: grey. Shown are means of results for 20 simulations. Light phase is indicated by yellow bar and dark phase indicated by black bar. Boxplots are dodged by 0.25 h in order to prevent overlap.

Flux within the GS/GOGAT cycle (Figure 7) was tightly linked to *PR*. For *GS* and *GOGAT* genotype and treatment effects were observed (treatment effect on *GS* and *GOGAT*: *p* < 2e-16; genotype effect on *GS*: *p* < 2e-16; genotype effect on *GOGAT*: *p* = 5.63e-14). The highest flux was obtained at ambient CO_2_ for *hpr1-1*, followed by Col-0 at ambient. For plants grown at eCO_2_, a slightly higher flux was observed for *hpr1-1* as compared to wildtype, supporting an earlier finding that PR takes place even at a CO_2_ concentration of 1,000 ppm (Kraemer et al., 2021a). In summary, the mutant showed a higher turnover in the GS/GOGAT cycle compared to the wildtype, and this turnover was decreased at eCO_2_. However, flux of *NR* (Figure 7C) behaved differently. *NR* flux was higher in Col-0 and rose at eCO_2_, revealing significant genotype and treatment effects (*p* = 5.66e-11 and *p* = 4.79e-07, respectively).

## Discussion

### Interaction of eCO_2_ and N-Assimilation

Several studies have pointed out that eCO_2_ decreases the N content of plant biomass (Bloom et al., 2014; Andrews et al., 2020). However, the underlying mechanism is unclear. Low stomatal conductance at high internal CO_2_ concentrations could reduce nitrate availability, but in contrast to observations for wheat under eCO_2_ (Del Pozo et al., 2007), we found no indications for reduced mineral content in *Arabidopsis* wildtype plants (Supplementary Image 3A). Although no consistent change of foliar nitrate was observed in the wildtype, it was significantly lower in the *hpr1-1* mutant especially under ambient CO_2_. This points to a restricted uptake capability in *hpr1-1*, probably because of energetic constraints (see below).

**Figure 8:**
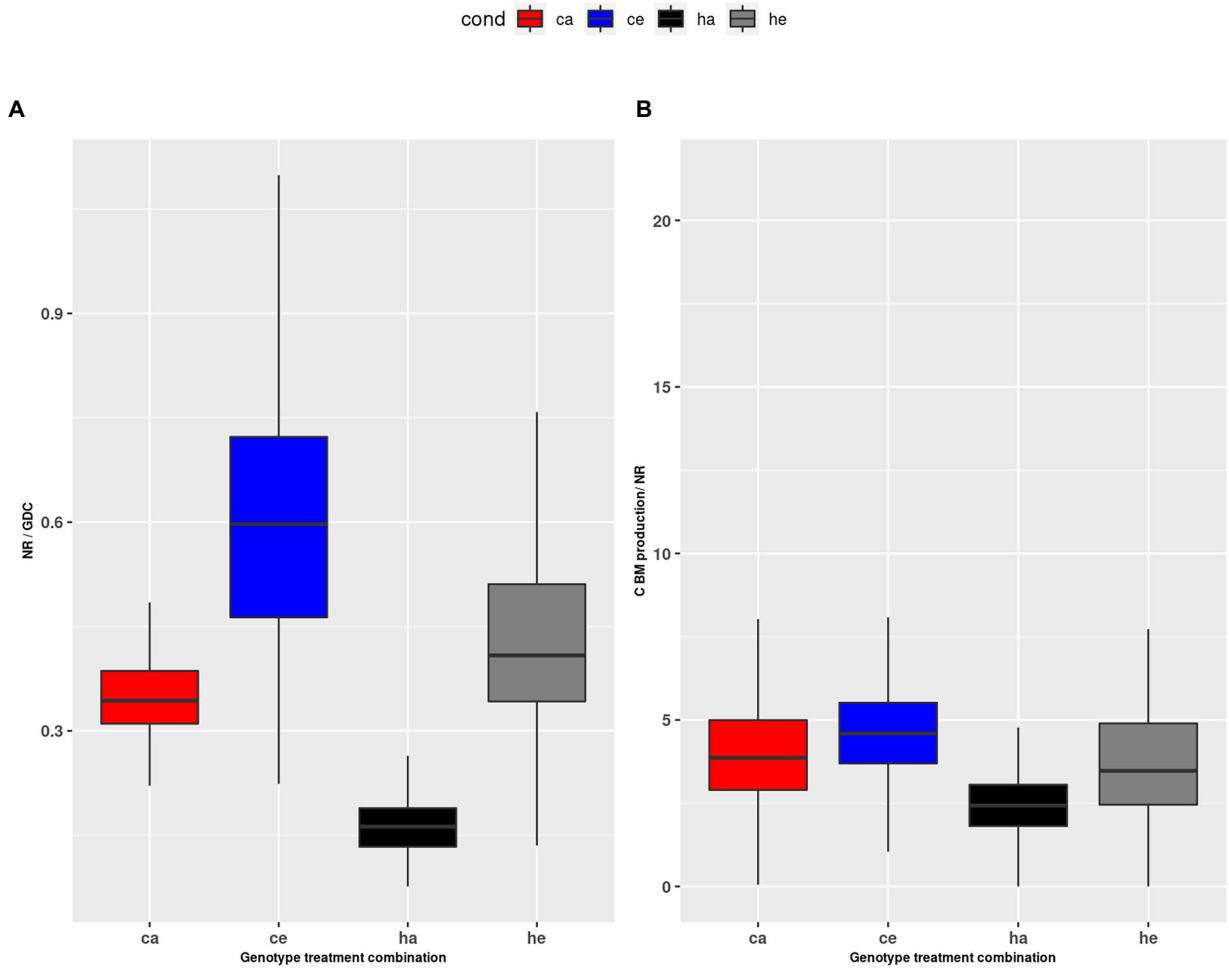
**(A)** Ratio of *NR* to *GDC* during the day, **(B)** ratio of HP2BMEXP to NR during the day. Col-0 ambient: red, Col-0 eCO_2_: blue; *hpr1-1* mutant ambient: black, *hpr1-1* mutant eCO_2_: grey. Data based on 20 simulations. Boxplots are dodged by 0.25 h in order to prevent overlap.

The alternative possibility that nitrate reduction was inhibited at eCO_2_ (Bloom et al., 2014; Zhao et al., 2021) was also not supported in the current study. We found that the v_max_ for NR increased at eCO_2_ (Figure 7C), and the calculated *NR* flux was higher at eCO_2_ in wildtype plants. The observation that *NR* was consistently higher in Col-0 as compared to the *hpr1-1* mutant, which had high levels of free AA, further argues against insufficient N supply. Thus, our results are in support of reports favoring a dilution of total N to accompany increased carbon fixation and biomass formation under eCO_2_ (Andrews et al., 2019). For eCO_2_, we found a higher ratio of *NR* to *GDC* (Figure 8A). Both fluxes feed into the pool of ammonium used for Gln synthesis. A low *GDC* contribution would reduce the load on the GS/GOGAT cycle, allowing a higher proportion of *de novo* N assimilation. Indeed, we calculated a lower flux for *GS/GOGAT* at eCO_2_ (Figures 7A,B), demonstrating a tight link between *PR* and *GS/GOGAT* turnover. This link has also been shown by Häusler et al. (1994) and Wallsgrove et al. (1987), who reported that barley mutants lacking GS activity suffered under photorespiratory conditions. In addition, it is known that even short incubations at eCO_2_ result in a reduction of GS and GOGAT activity (Guo et al., 2013; Wu et al., 2020). Our finding that the ratio of NR to GDC activity was elevated at eCO_2_ in the wildtype as well as the mutant indicates that the GS/GOGAT cycle contained more newly assimilated N at eCO_2_, and this further argues against N starvation at eCO_2_.

In a previous study comparing plants exposed to eCO_2_ either short or long term, we revealed that PR allows deposition of already assimilated N in low carbon-containing AA like Gly and Ser, thus providing carbon skeletons for *de novo* N assimilation (Kraemer et al., 2021a). While this can explain photosynthetic acclimation to long-term eCO_2_, which results in reduced *PS* as well as *NR* rate, it does not explain an imbalance in the ratio of both fluxes. Whether sufficient N for the production of biomass was assimilated at eCO_2_ should be reflected in the ratio of the carbon flux HP2BMEXP and the *de novo* N fixation by NR. Figure 8B shows a significant but not dramatic increase of this ratio at eCO_2_. Considering the increased NR flux, this indicates that carbon assimilation was even more stimulated than N fixation at eCO_2_, which would support the concept of N dilution at eCO_2_ (Wong, 1990; Kuehny et al., 1991; Gifford et al., 2000; Taub and Wang, 2008). This is also substantiated by the strongly increased starch levels (Supplementary Image 3B). In the mutant, however, we and others (Timm et al., 2008) found a large pool of free amino acids (Figures 1, 2), which apparently contradicts an increased C/N ratio.

### Phenotype of the *hpr1-1* Mutant

As mentioned above, an energetic constraint could be responsible for the metabolic disturbance in the mutant. As shown in Figure 7, the fluxes for *GS* and *GOGAT* are increased in *hpr1-1* as compared to wildtype especially at ambient CO_2_. Thus, large amounts of ATP and reduced ferredoxin are required to sustain synthesis of Glu, which is needed for the removal of glyoxylate. Figure 8A shows that the *NR*-to-*GDC* ratio was lowest for *hpr1-1* at ambient, thus indicating futile cycling of ammonium without net gain of biomass.

While a loss of cellular energy could explain the differences in biomass formation of wildtype and mutant at different CO_2_ concentrations, which have already been described (Timm et al., 2008), futile cycling of already assimilated ammonium could be regarded as conflicting with high levels of amino acids in the mutant. Accumulation of starch, carboxylates and AA in the mutant could simply result from a slow growth rate as suggested by Génard et al. (2014), but than a reason different from resource limitation must underly stunted growth and chlorotic phenotype of *hpr1-1*.

A possible explanation could be poisoning by photorespiratory intermediates such as phosphoglycolate, glycolate or glyoxylate (Anderson, 1971; Dellero et al., 2016). As can be seen in Figure 6 the *GDC* and *SHMT* fluxes are strongly increased in *hpr1-1* at ambient CO_2_. But still the *HPR* flux is substantially extended into the night. This shows that even after light-off photorespiratory intermediates had to be recycled, which were not metabolized during the day in spite of the increased GDC and SHMT activity. It can thus be assumed that phosphoglycolate accumulates during the day, and this would strongly inhibit triose-phosphate isomerase (Anderson, 1971), which would in turn block carbon assimilation in the Calvin-Benson cycle. But how should a bottleneck in the last step of the PR pathway cause accumulation of the early metabolites? As we have already described (Kraemer et al., 2021a), simulations of a metabolic model for the *hpr1-1* mutant pointed to an additional source of glycolate, which is independent from oxygenation of ribulose-bisphosphate. A very likely candidate is the non-enzymatic oxidation of hydroxypyruvate by H_2_O_2_ in the peroxisome, which yields glycolate (Walton and Butt, 1981). This reaction is promoted by the large amount of Ser in the mutant at ambient CO_2_. Bao et al. (2021) showed that already a fourfold increase of Ser levels in the catalase mutant *cat2* caused a significant increase in hydroxypyruvate decarboxylation. Considering that Ser levels in *hpr1-1* were about tenfold higher than wildtype at the end of the day, it is highly likely that non-enzymatic decarboxylation of hydroxypyruvate takes place in *hpr1-1*. Thus, in the *hpr1-1* mutant more glycolate is produced in relation to photosynthetic carbon acquisition as compared to the wildtype. This would not only increase the probability of a toxic effect, but also cause additional loss of assimilated carbon in the form of CO_2_.

Besides toxication by photorespiratory intermediates, it would also be possible that an increased level of ammonium (Figure 1C) could interfere with ATP production, because it is in equilibrium with ammonia that acts as an uncoupling agent of ATP synthesis.

Finally, the metabolic bottleneck created by the *hpr1-1* mutation caused large amounts of C and N being bound in the form of Gly and Ser. As a consequence, the equilibrium of free amino-acids, brought about by transamination reactions, might be severely disturbed, and this could interfere with protein synthesis in the shoot as well as the supply of the roots and other sinks with AA. Not only N but also C compounds show altered distributions in *hpr1-1* (Supplementary Image 1; Figure 2). Especially the carboxylates Cit, malate and fumarate, were significantly enriched, while glucose was reduced in *hpr1-1*. We did not detect a drop in foliar sucrose content, but the lowered nitrate content of *hpr1-1* shoots in ambient CO_2_ might indicate low carbon supply to the root system. The low glucose level in leaves could result from enhanced use in the pentose-phosphate pathway as suggested by Li et al. (2019), who reported that high pentose-phosphate pathway activity could provide additional CO_2_ that would alleviate the PR syndrome, but restrict biomass formation. A combination of the above described effects could contributes to the mutant phenotype of *hpr1-1*.

## Conclusion

We showed that the *hpr1-1* mutant suffers from several limitations. Besides a high demand for ATP and reducing equivalents for the increased turnover of the GS/GOGAT cycle, a possible toxication by photorespiratory intermediates or NH_4_^+^ could interfere with biomass formation, and an unfavorable redistribution of N and C compounds could contribute to restricted biomass production. Our study confirms a tight link between PR and the GS/GOGAT cycle and adds to our understanding of how plant N assimilation is affected by eCO_2_.

## Data Availability Statement

The raw data supporting the conclusions of this article can be found in the Supplementary Material, further inquiries can be directed to the corresponding author.

## Author Contributions

KK and AH designed the study and wrote the manuscript. KK developed the model. KK and JB conducted the experiments. All authors contributed to the article and approved the submitted version.

## Funding

KK was supported by a scholarship “Landesgraduiertenförderung (LGF)” of the Federal State of Baden-Wuerttemberg (Germany).

## Conflict of Interest

The authors declare that the research was conducted in the absence of any commercial or financial relationships that could be construed as a potential conflict of interest.

## Publisher’s Note

All claims expressed in this article are solely those of the authors and do not necessarily represent those of their affiliated organizations, or those of the publisher, the editors and the reviewers. Any product that may be evaluated in this article, or claim that may be made by its manufacturer, is not guaranteed or endorsed by the publisher.
