# Supplementary material for: Interaction of Nitrate Assimilation and Photorespiration at Elevated CO2
Source: Front Plant Sci. 2022 Jul 1;13:897924. doi: 10.3389/fpls.2022.897924 (PMC9284316; doi:10.3389/fpls.2022.897924)
Supplement: Supplementary file 1 [file Data_Sheet_1.pdf]

GDC

$GDC = \frac{vmax_{GDC}*Gly}{km_{GDC}+Gly}$

SHMT

$GDC = \frac{vmax_{SHMT}*Gly}{km_{SHMT}+Gly}$

if (SHMT > GDC) { SHMT = GDC }

HPR

$HPR = \frac{vmax_{HPR}*Ser}{km_{HPR}+Ser}$

if(HPR > (PR/2.)) { HPR = PR/2. }

GS

$GS = \frac{vmax_{GS}*Glu*NH_4^+}{km_{Glu}*km_{NH_4^+}+km_{Glu}*NH_4^+ +km_{NH_4^+}*Glu+Glu*NH_4^+}$

if(t > 8.) { GS = GS*gs_inactivation }

GOGAT

$GOGAT = \frac{vmax_{GOGAT}*Gln*\alpha KG}{km_{\alpha KG}*km_{Gln}+km_{\alpha KG}*Gln+km_{Gln}*\alpha KG+Gln*\alpha KG}$

NR

$NR = \frac{vmax_{NR}*NO_3^-}{km_{NR}+NO_3^-}$
