# Supplementary material for: Interaction of Nitrate Assimilation and Photorespiration at Elevated CO2
Source: Front Plant Sci. 2022 Jul 1;13:897924. doi: 10.3389/fpls.2022.897924 (PMC9284316; doi:10.3389/fpls.2022.897924)
Supplement: Supplementary file 2 [file Data_Sheet_2.pdf]

```cpp
typedef std::vector<double> VD;

void model(double t, double PS, double PR, double STARCH, double NIT, double RESPIRATION, VD&
params, VD& states) {

  /*
  PS is µmol C6 / (g*h)
  PR is µmol C2 / (g*h)
  RESPIRATION is µmol C6 / (g*h)
  */

  // Extract parameters

  // HPR
  double vmax_hpr = params[0];
  double km_hpr = params[1];

  // NR
  double vmax_nr = params[2];
  double km_nr = params[3];

  // GS
  double vmax_gs = params[4];
  double km_gs_GLU = params[5];
  double km_gs_AMMONIUM = params[6];

  // GOGAT
  double vmax_gogat = params[7];
  double km_gogat_GLN = params[8];
  double km_gogat_KG = params[9];

  // remaining parameters
  double alpha = params[10];
  double hp_to_bmexp = params[11];
  double hp_to_mf = params[12];
  double mf_to_cit = params[13];
  double cit_to_mf = params[14];
  double cit_to_akg = params[15];
  double aa_to_bmexp = params[16];
  double ser_to_aa = params[17];
  double correct = params[18];
  double gdc_vmax = params[19];
  double shmt_vmax = params[20];
  double km_gly_gdc = params[21];
  double km_gly_shmt = params[22];
  double gs_inactivation = params[23];

  // Extracting states
  double GLY = states[0];
  double SER = states[1];
  double AMMONIUM = states[2];
  double AA = states[3];
  double GLU = states[4];
  double GLN = states[5];
  double HP = states[6];
  double MF = states[7];
  double CIT = states[8];
  double KG = states[9];

  // only 5.0425% Cytosol  Koffler2013
  NIT = NIT*0.050425;

  // Enzyme kinetics
  double HPR = (vmax_hpr*SER) / (km_hpr + SER);
  double NR = (vmax_nr*NIT) / (km_nr + NIT);
```

```
  double GOGAT = (vmax_gogat*GLN*KG) / (km_gogat_KG*km_gogat_GLN + km_gogat_KG*GLN +
km_gogat_GLN*KG + GLN*KG);
  double GS = (vmax_gs*GLU*AMMONIUM) / (km_gs_GLU*km_gs_AMMONIUM + km_gs_GLU*AMMONIUM +
km_gs_AMMONIUM*GLU + GLU*AMMONIUM);

  double GDC = (gdc_vmax*GLY) / (km_gly_gdc + GLY);

  //shmt_vmax = gdc_vmax;
  //km_gly_shmt = km_gly_gdc;
  double SHMT = (shmt_vmax*GLY) / (km_gly_shmt + GLY);

  if(SHMT > GDC) {
    SHMT = GDC;
  }

  // factors:
  // c2 flux to c6 flux
  double factor0 = 2./6.;
  // AA C6 N1.23
  // ca. 14.3197% of proteom consists of Ser
  double factor1 = 0.143197;
  // C6 to C5
  double factor2 = 6./5.;

  // nightly flux in gly & maximal HPR rate
  double PR_new = PR;
  if(t > 8.) {
  //  PR_new = PR + alpha;
  }
  PR_new = PR + alpha;
  if(HPR > (PR_new/2.)) {
    HPR = PR_new/2.;
  }

  /*
  respiration is reduced by photorespiration
  But only if GDC*GLY is smaller than respiration
  */
  double RESPIRATION_new = RESPIRATION + (GDC)*factor0;
  if(RESPIRATION_new > 0.) {
    RESPIRATION_new = 0.;
  }

  if( (t < 8.) &&  (RESPIRATION_new < 0.) ) {
      PS = PS - RESPIRATION_new;
  }

  /*
  regulation of gs and nr during night
  flux from ser to aa only during night
  */
  double ser_to_aa_new = 0.;
  if(t > 8.) {
      ser_to_aa_new = ser_to_aa;
      GS = GS*gs_inactivation;
  }

  double GLU_TO_AA = NR*correct;

  /*
  N balance in order to calculate flux from Glu to aKG
  */
  double GLU_GLYOXYLATE_AT = PR_new - HPR;
  if(GLU_GLYOXYLATE_AT < 0.) {
    GLU_GLYOXYLATE_AT = 0.;
```

```
  }

  // ODE System
  double ddtGLY = states[0] = PR_new - GDC - SHMT;
  double ddtSER = states[1] = (1./3.)*GDC + (2./3.)*SHMT - HPR - SER*ser_to_aa_new -
AA*aa_to_bmexp*factor1;
  double ddtAMMONIUM = states[2] = GDC + NR - GS;
  double ddtAA = states[3] = GLU_TO_AA - AA*aa_to_bmexp + (SER*ser_to_aa_new)/2.;
  double ddtGLU = states[4] = 2.*GOGAT - GS - GLU_TO_AA - GLU_GLYOXYLATE_AT;
  double ddtGLN = states[5] = GS - GOGAT;
  double ddtHP = states[6] = PS + HPR/2. - HP*hp_to_bmexp - HP*hp_to_mf - STARCH;
  double ddtMF = states[7] = HP*hp_to_mf + RESPIRATION_new - MF*mf_to_cit + CIT*cit_to_mf;
  double ddtCIT = states[8] = MF*mf_to_cit - CIT*cit_to_mf - CIT*cit_to_akg;
  double ddtKG = states[9] = factor2*CIT*cit_to_akg + GLU_GLYOXYLATE_AT - GOGAT;
  double ddtbilanz = states[10] = PR_new/3 + PS + RESPIRATION_new -HP*hp_to_bmexp -
AA*aa_to_bmexp*(6./5.);

}
```
