# Supplementary material for: Interaction of Nitrate Assimilation and Photorespiration at Elevated CO2
Source: Front Plant Sci. 2022 Jul 1;13:897924. doi: 10.3389/fpls.2022.897924 (PMC9284316; doi:10.3389/fpls.2022.897924)
Supplement: Supplementary file 3 [file Image_1.pdf]

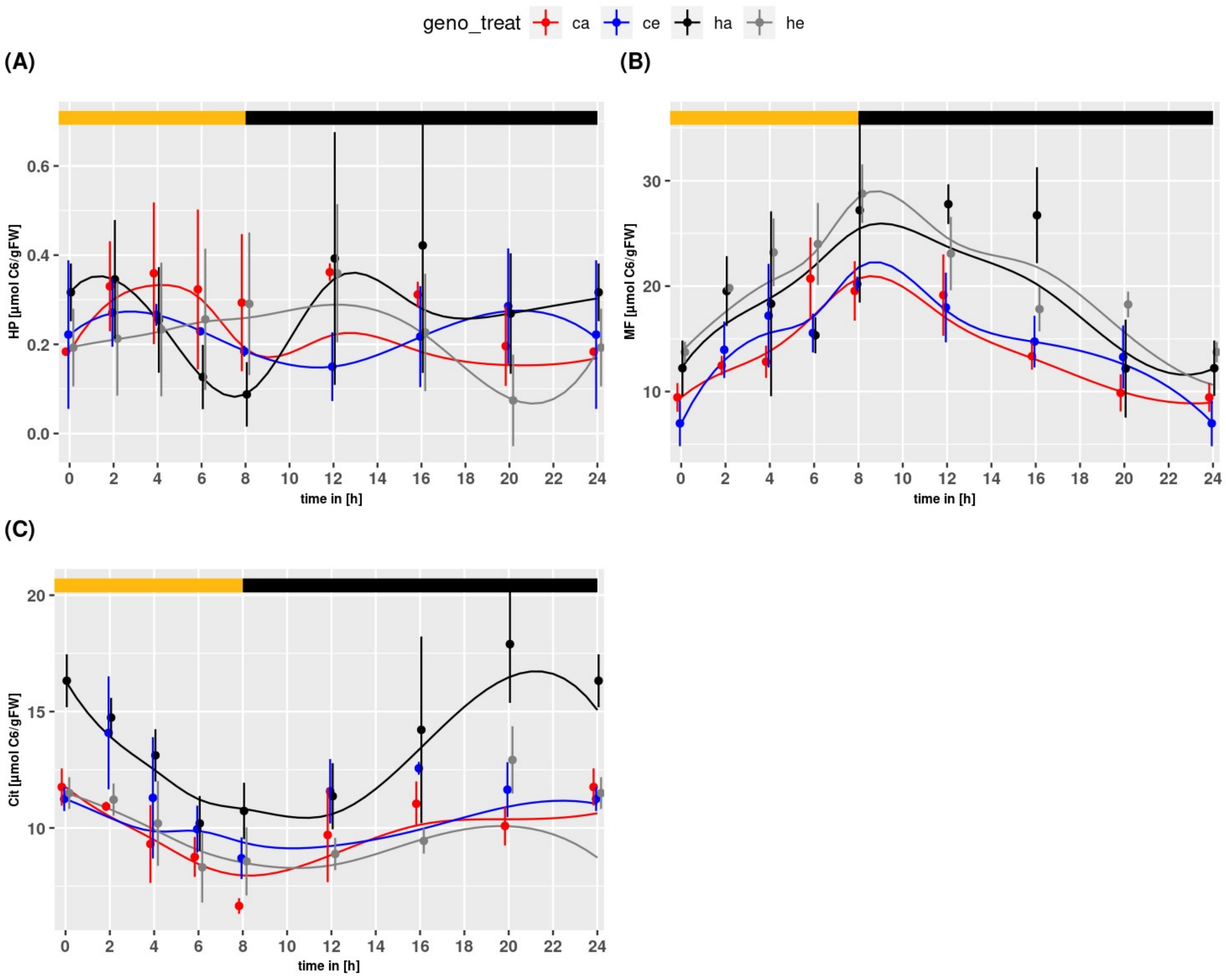

Figure S1: Diurnal course of (a) HP, (b) MF, and Cit (c). In red the wildtype and in blue the hpr1-1 mutant. Dashed lines and triangles represent elevated CO_2 concentrations. Continous lines and closed circles represent ambient CO_2 concentrations. Dots or triangles represent mean of measured values (n = 5 per group). Bares indicate the error of measurements. Line represent the mean of 20 simulations. Light phase indicated by yellow bar and dark phase indicated by black bar.
