# Supplementary material for: Interaction of Nitrate Assimilation and Photorespiration at Elevated CO2
Source: Front Plant Sci. 2022 Jul 1;13:897924. doi: 10.3389/fpls.2022.897924 (PMC9284316; doi:10.3389/fpls.2022.897924)
Supplement: Supplementary file 4 [file Image_2.pdf]

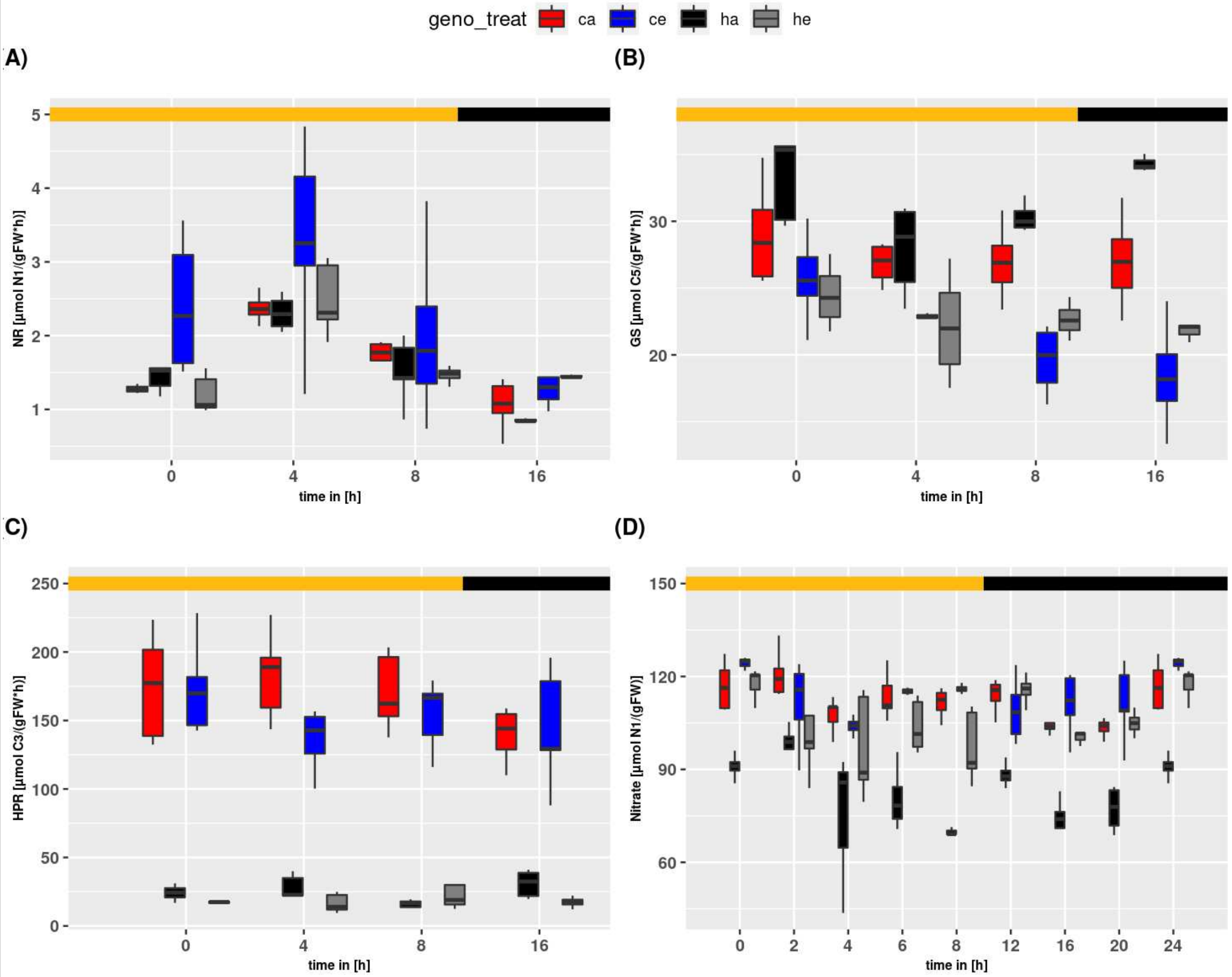

Figure S2: Diurnal course of measured activities (at least n = 5 per group) for (a) NR, (b) GS, and HPR (c). In red the wildtype and in blue the hpr1-1 mutant. Dashed boxplots represent ambient CO_2 concentrations. Dotted boxplots represent elevated CO_2 concentrations. Light phase indicated by yellow bar and dark phase indicated by black bar. Boxplots are dodged by 0.25 h in order to prevent overlapping.
