# Supplementary figures and images for: Interaction of Nitrate Assimilation and Photorespiration at Elevated CO2
Source: Front Plant Sci. 2022 Jul 1;13:897924. doi: 10.3389/fpls.2022.897924 (PMC9284316; doi:10.3389/fpls.2022.897924)

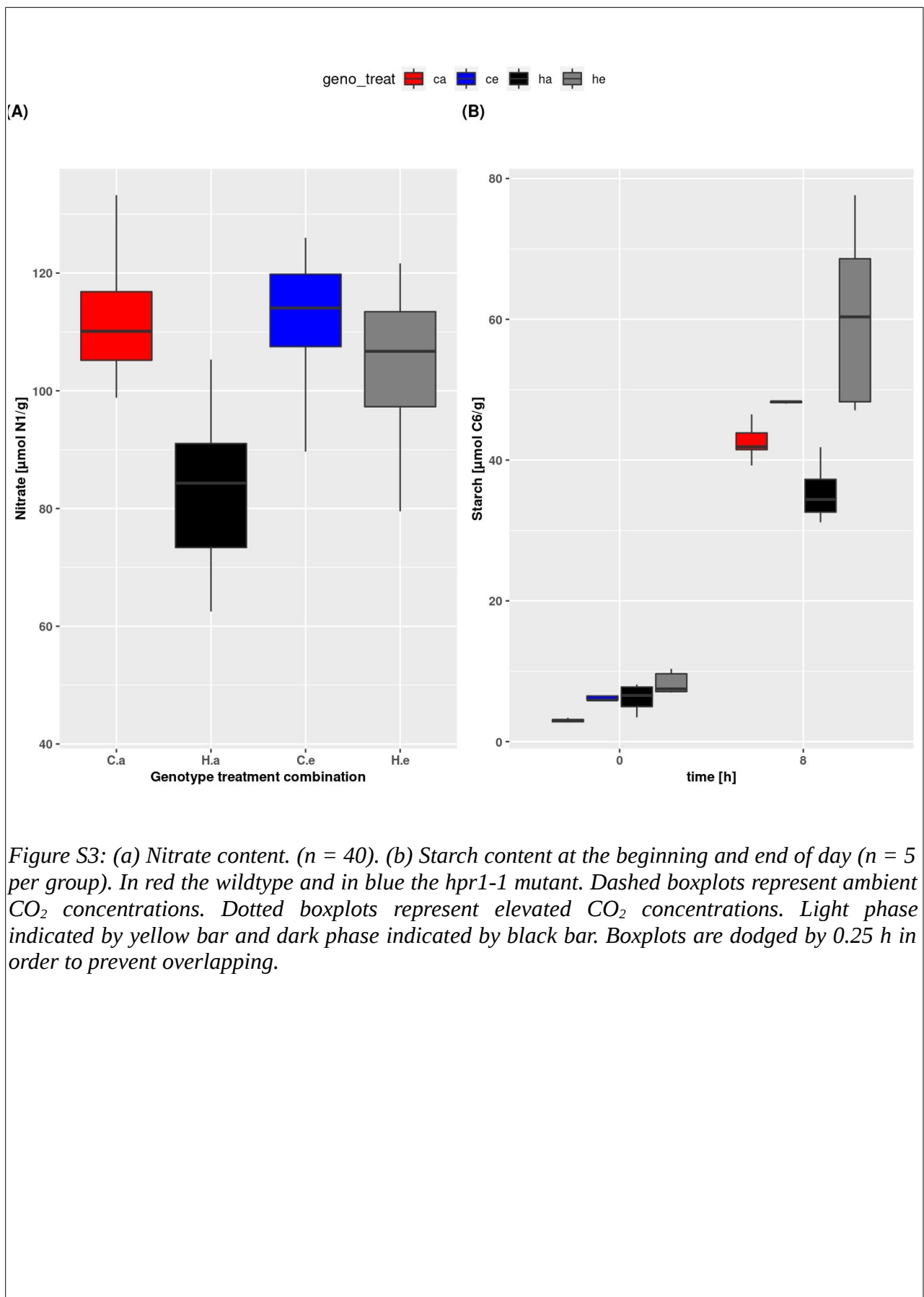

Supplement: Supplementary file 5 [file Image_3.pdf]
